# A simple parasitological technique to increase detection of *Strongyloides stercoralis* in Bolivian primary health care system

**DOI:** 10.1186/s12875-022-01888-4

**Published:** 2022-11-17

**Authors:** Ivana Camacho-Alvarez, Elia M. Chavez-Mamani, Goyens Philippe, Jenny M. Luizaga-López, Mary Cruz Torrico, Laurent Gétaz, Frédérique Jacobs

**Affiliations:** 1 Medical Sciences Université Libre de Bruxelles (Belgium); Public Health and Epidemiology, University of San Simón (Bolivia), Cochabamba, Bolivia; 2 Biomedical and Social Research Institute, Medicine Faculty, University of San Simón, Cochabamba, Bolivia; 3 Nutrition and Metabolism Unit, Department of Pediatrics, Hôpital Universitaire des Enfants Reine Fabiola HUDERF, Brussels, Belgium; 4 Biomedical and Social Research Institute, University of San Simón, Cochabamba, Bolivia; 5 Medical Research Laboratory, Parasitology, Medicine Faculty, University of San Simón, Cochabamba, Bolivia; 6 Division of Tropical and Humanitarian Medicine, Geneva University Hospitals and University of Geneva, Geneva, Switzerland; 7 Infectious Diseases Department at Université Libre de Bruxelles, CUB-Erasme, Brussels, Belgium

**Keywords:** Neglected Tropical Disease, *Strongyloides Stercoralis*, Diagnosis, Baermann Technique, Primary Health Care

## Abstract

**Background:**

*Strongyloides stercoralis* is widespread; however, there is limited information on its prevalence owing to laboratory underestimation and low clinical manifestations. The Baermann method and agar culture stand out among the parasitological techniques. *Strongyloides stercoralis* is present in Bolivia, but its prevalence in children remains unknown. The objective of this study was to estimate the applicability of simple parasitological techniques to increase the detection of this parasite in children living in the tropics.

**Methods:**

This cross-sectional study was conducted in a tropical village in Cochabamba, Bolivia. Participants were 304 children aged 5 – 12 years who provided stool samples for different parasitological analyses (direct examination, Ritchie, Baermann, and Dancescu techniques), and their parents provided informed consent.

**Results:**

Up to 64.8% of pathogenic parasites were detected using the modified Ritchie method. The Baermann technique identified 17.8% of *Strongyloides stercoralis* cases, and a high sensitivity with respect to the Baermann technique was only for the Dancescu technique (75.9%) that is also specific for *Strongyloides stercoralis*, followed by 66.7% for the modified Ritchie technique, which is used in second-line care.

**Discussion:**

The Baermann technique is the best parasitological option for improving *Strongyloides stercoralis* diagnosis in the first-line care of the Primary Health Care System. A particular cycle of reinfection, combined with the environment and some other risk factors are related with persistence. Control is difficult without a proper diagnosis, and the Baermann technique is an approach to the solution. We conclude that with a high suspicion of the presence of *Strongyloides stercoralis*, the use of the Baermann technique is strongly recommended as support for direct examination in primary health care systems especially in tropical areas.

**Supplementary Information:**

The online version contains supplementary material available at 10.1186/s12875-022-01888-4.

## Introduction

The distribution of *Strongyloides stercoralis* is widespread worldwide, especially in Latin America, because of the favourable conditions offered by tropical environments. However, very little information is available on its prevalence, which is underestimated because sensitive methods are not widely used for diagnosis in the first approach [1, 2].

A frequent reason for the underestimation of *S. stercoralis* is that the symptoms are absent, non-specific, or very mild when the parasite burden is low in an initial infection (anal itching, abdominal pain, or respiratory discomfort) [3, 4]. The risk of infection in endemic areas with poor sanitary conditions varies with age, being more vulnerable children than adults. In children, several clinical manifestations have been found, including intestinal, dermatological, or nutritional manifestations that vary according to the parasite load and time of infection, since *S. stercoralis* can complete its life cycle in the same host, facilitating the maintenance and spread of infection. Thus, long-term chronic effects will be associated with malnutrition whose effects also affect physical and cognitive development [5–8].

Currently, several diagnostic methods are available for *S. stercolaris* at the parasitological, serological, and molecular levels [9, 10], the use of which also depends on the complexity of the level of care to which the user has access. Among the parasitological techniques, the Baermann method and agar culture stand out, as their performance is much better than that of a simple direct examination and even better than that of the formalin-ether concentration technique (Ritchie) [10, 11]. Serological and molecular studies have shown significantly increased diagnostic sensitivity; however, their specificity remains to be evaluated [9, 10, 12].

In basic public health measures, especially in developing countries, early diagnosis is sought for early treatment in first-line units, which must be considered the most important part of the primary health care (PHC) system for making the first contact with individuals in their communities. However, health resources are mostly invested in high-complexity health services, which usually leave first-line care with challenges and improvements to gradually accomplish, requiring low-cost and easy-to-implement services [13, 14]. Normally, in the PHC system from Bolivia, for the diagnosis of intestinal parasitic infections, direct examination in first-line units and the Ritchie technique in second-line units are used because they are well-standardized. Other techniques, such as Baermann and Dancescu, are more specific for S. stercoralis, but are less requested in general practice because of the lack of experience or training of medical or laboratory personnel and/or lack of supplies and materials [15–17]. Techniques of greater complexity, serological or molecular, are used in specialised care or research centres because of the required operating and training costs, which cannot be covered by the public health system [16].

*S. stercoralis* is very prevalent in Bolivia, especially in tropical areas, where conditions are favourable for its development [3, 18]. Its prevalence in Bolivian children under 12 years remains unknown. Children between 5 and 12 years of age are also considered a neglected population in the Bolivian PHC system owing to a lack of specific insurance [14]. Soil transmitted helminths (STHs) are underestimated, particularly *for S. stercoralis*, because of their difficulty in detection, as reported by other authors [1, 19, 20]. This study aimed to estimate the applicability of simple parasitological techniques for the detection of *S. stercoralis* in children from tropical endemic areas under basic laboratory conditions for primary health care, simulating a first-line centre.

## Methods

### Study region

The study was conducted in Shinahota village (17°0S, 65°15 W W), 183 km northeast of the tropical area of Cochabamba, from June to July 2017. The environmental conditions correspond to tropical areas, with humidity ranging between 50 and 89%, temperatures ranging between 16 and 25 °C, and lows of 3 – 12 °C. Shinahota has an altitude of 267 m. This village was selected on the basis of its location in a tropical area considered vulnerable to STH, willingness to participate in the population, and approval of the school principal to set up a laboratory and workspace for sample processing inside the school.

### Subjects

The participants were 304 children from two local elementary schools (Germán Busch A and Germán Busch B) with an age range of 5 – 12 years. Participation was voluntary and induced through a previous informative session with parents and professors on the prevention and detection of parasitic infections.

### Stool sample collection

Data were collected using a laboratory logbook for sample identification and a basic survey to detect risk factors associated with the presence of parasites, especially *S. stercoralis*. Participants were requested that the stool sample be as recent as possible (no more than 2 hours after collection) for laboratory analysis. Sterile bottles were provided, and recommendations were provided to parents for the sample to be equivalent to the size of the walnut.

### Parasitological analysis

Equipment to reproduce the basic conditions of a primary healthcare laboratory was installed in one of the schools to facilitate sample collection and delivery [16]. Stool samples were received daily in the schools, and each bottle was properly identified by the name and age of the patient, which were analysed using four methods: direct examination [21], Baermann technique, modified Ritchie technique [11], and carbon culture, Dancescu [22] which provides the appropriate conditions for *Strongyloides stercoralis* larvae for the development of their free life cycle.

**Fig. 1:**
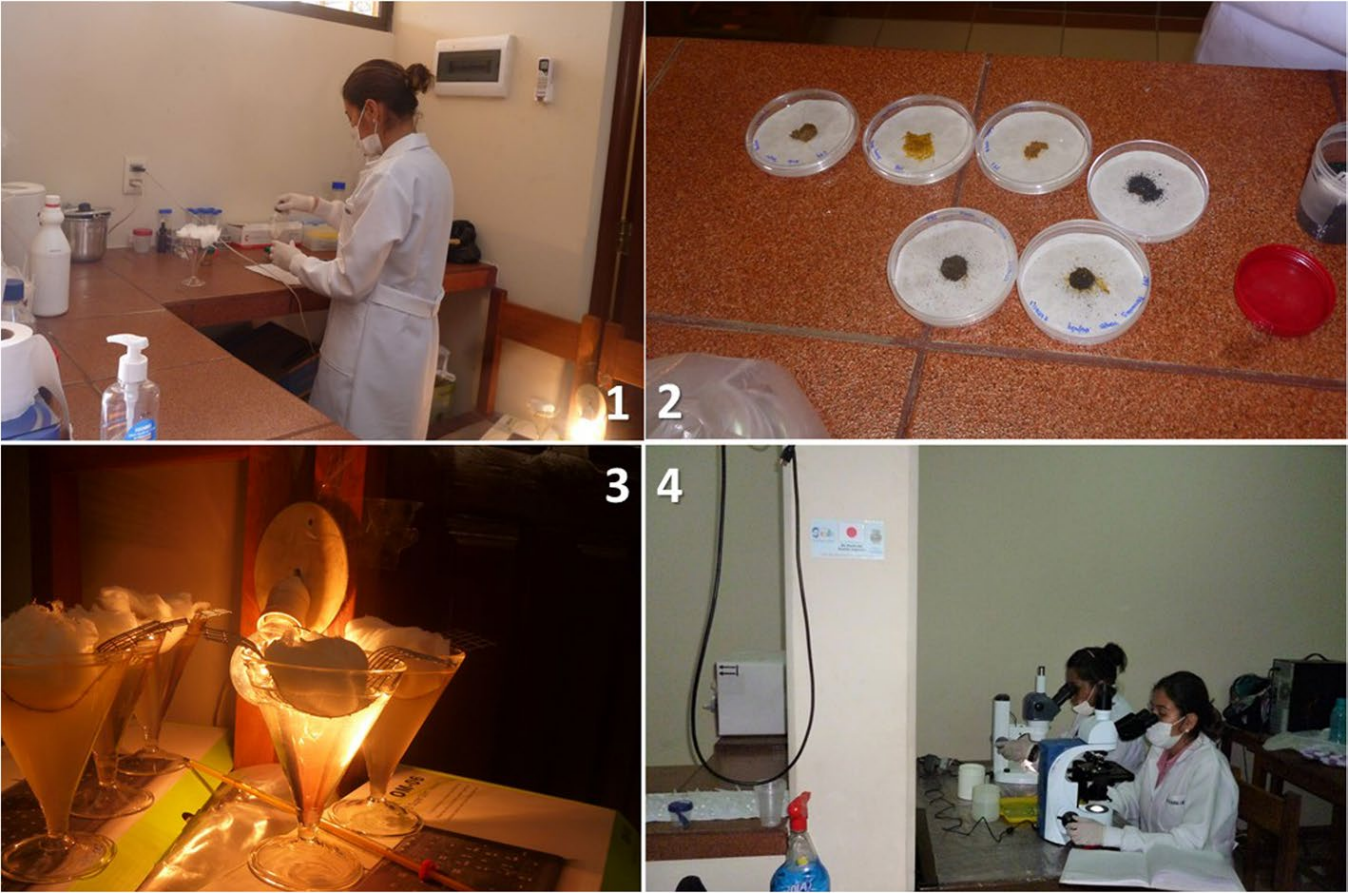
Field sample processing replicating first-line care laboratory conditions 1._Preparation of sample for Baermann technique and direct examination; 2. Preparation of plates for Dancescu technique; 3. Adaptation of Baermann technique for first level in the fieldwork and 4. On-site sample analysis

Direct examination is currently the only first-line technique for PHC in parasitology studies. The Ritchie technique is the technique par excellence used in the second line, and the Baermann and Dancescu techniques are used only upon specific requests for research in parasitology [16, 17]. For our study, the Baermann and Dancescu techniques were standardised and adapted for use in first-line care, and their conditions were reproduced in the fieldwork to improve their performance under these conditions. At the time of sample delivery, direct examination, the Baermann technique, and preparation of the plates for the Dancescu technique were performed immediately, ensuring that the quality of the sample was not lost due to desiccation (Fig. 1). For the direct examination, 2 g of faecal sample is taken, two fields are formed on a glass plate by mixing 1 g with 0.85% saline solution and 1 g with 4% Lugol, immediately taken to the microscope. Then, for the Baermann method, 5 g of sample is taken on a gauze mesh, which is partially immersed in a conical beaker with physiological solution at 26 °C and rests for at least 30 min near the source that maintains the heat in the lower part of the beaker. Concentration is expected by migration in the lower, narrower part of the beaker, from which 3 ml is taken on a special glass plate for direct reading under the microscope. In the modified Baermann technique, incandescent bulbs were used near the sedimentation vessels to maintain a suitable temperature of 37 °C, necessary for optimal results, verified and measured with a mercury thermometer [23].

Meanwhile, for Dancescu, a mixture with charcoal is prepared with another 5 g of the sample in a Petri dish with moistened filter paper, then sealed in plastic containers to maintain humidity, temperature, avoid contamination and left to stand for 4 to 6 days. Readings are taken after the 4th day to avoid the development of other hookworm larvae and thus false positives. An inverted microscope was used, which is an unusual item in first level laboratories; however, it was implemented in this study to improve the results [22] (Fig. 1). Once each faecal sample was processed, the residues were preserved in 10% formalin and transported to the Parasitology Laboratory of the University to apply the modified Ritchie technique.

For each method used, positive samples were confirmed by microscopy at 400x magnification in basic and university laboratories. The pathogenic parasites (PP) identified in direct examination and modified Ritchie were classified into two groups, protozoa including: *Giardia duodenalis*and *Entamoeba histolytica*; and helminths that includes: *Strongyloides stercoralis, Ascaris lumbricoides, Trichuris trichiura*, Hookworm, *Enterobius vermicularis* and *Hymenolepis nana*. The Baerman and Dancescu methods only help us to identify *S. stercoralis*. The samples were disposed of after inactivation with calcium oxide (quicklime) in red bags according to the Bolivian national recommendations for the disposal of faecal samples [23].

### Ethics statement

This study was reviewed and approved by the **Bioethics Committee of the Faculty of Medicine “Aurelio Melean”** of San Simón University (MTG 30.06.2017). Prior to sample collection, parents and their children were given an oral briefing on the study objectives and methodology. Participants were informed that their participation was voluntary, and that their identities and personal information would be kept confidential. If they agreed to participate, consent was obtained from their parents or guardians, who are usually heads of the family. All the participants provided written informed consent. For illiterate participants, consent was obtained from their thumbprint on an informed consent form.

Upon completion of the consent form, participants were asked to answer a pre-designed questionnaire to collect information on the demographic data. The container with the serial number for stool sample collection was provided to parent(s) or guardian(s) of children who agreed to participate in the study. All children with parasitic infections were treated once the results were confirmed by four parasitological examinations.

### Statistical analysis

Statistical analysis was performed using SPSS software (version 22.0) for Windows. Descriptive data were expressed as percentages and frequencies. Means and standard deviations (SDs) were used to represent normally distributed variables. Comparisons of parasitological methods and differences in the time for positive results for *S. stercoralis* were performed using MedCalc version 20.008. Statistical significance was set at *P* ≤ 0.05, for univariate and multivariate analyses.

## Results

**Fig. 2:**
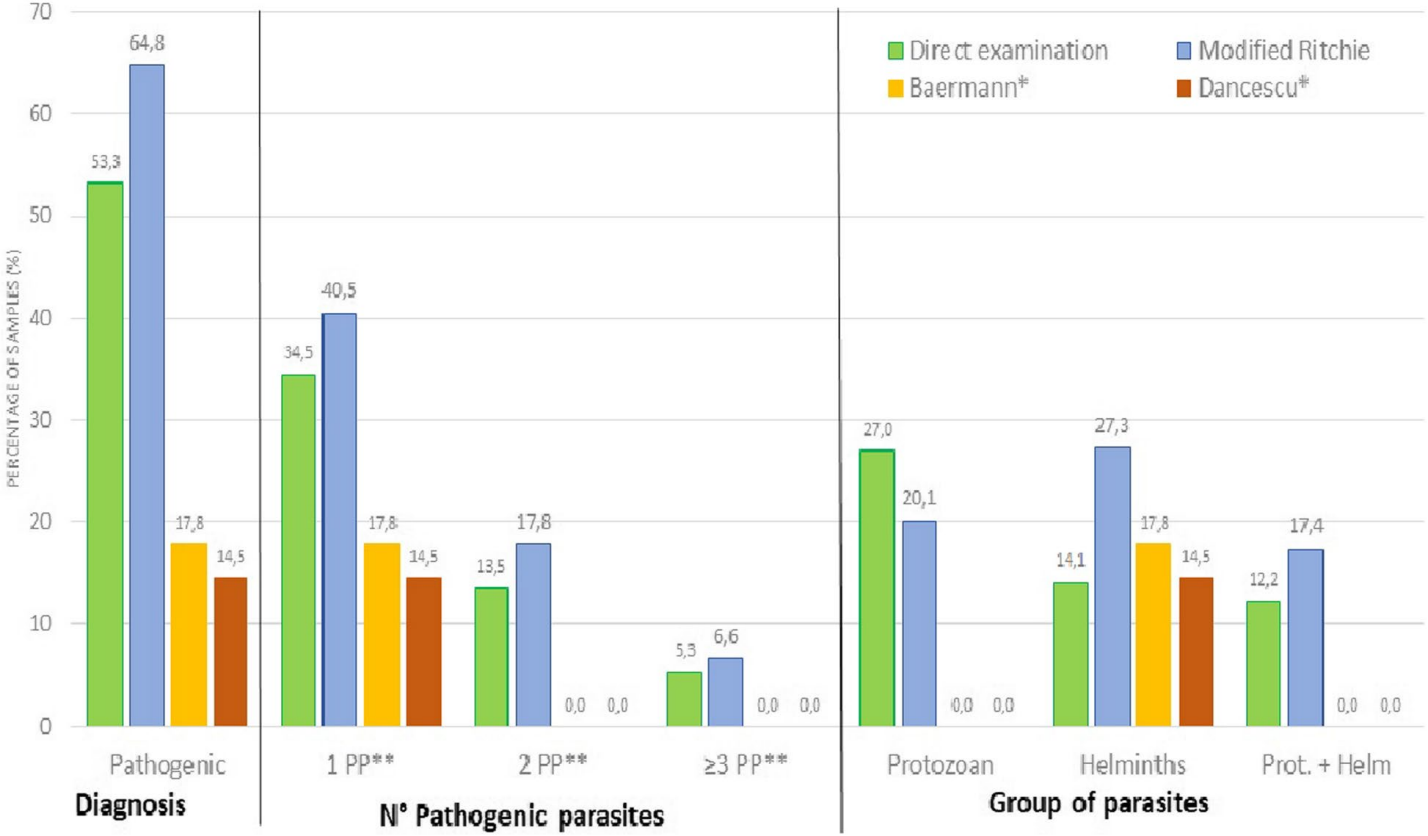
Detection of intestinal parasites using four parasitological techniques in primary health care in children from Bolivian tropical areas. *Specific methods for Strongyloides stercoralis. **PP: Pathogenic parasite

Our sample of 304 children was comprised of 156 (51.3%) males and 148 (48.7%) females, with a mean age of 7.9 years (SD ± 1.8). Fig. 2 shows the comparative performances of the four techniques. The presence of parasites was detectable in equal proportions by direct examination and the Ritchie technique (83.2%), as well as the number of parasites that could be identified with both techniques (up to six in the case of direct examination). However, of the parasites identified, 53.3% (N: 162) were classified as pathogenic by direct examination and 64.9% (N: 197) were classified using the Ritchie method.

Regarding the classification of parasite groups or combinations, direct examination was better at identifying protozoa (27%, N: 82), while the Ritchie method identified more helminths (27.3%, N: 83). These statistically significant differences show the Ritchie method to be very useful for the identification of multiparasitized samples. In contrast, the Baermann and Dancescu techniques are specifically used for the detection of *S. stercoralis*, they only show numbers related to the presence or absence of it and very exceptionally could allow the identification of other parasites.

**Fig. 3:**
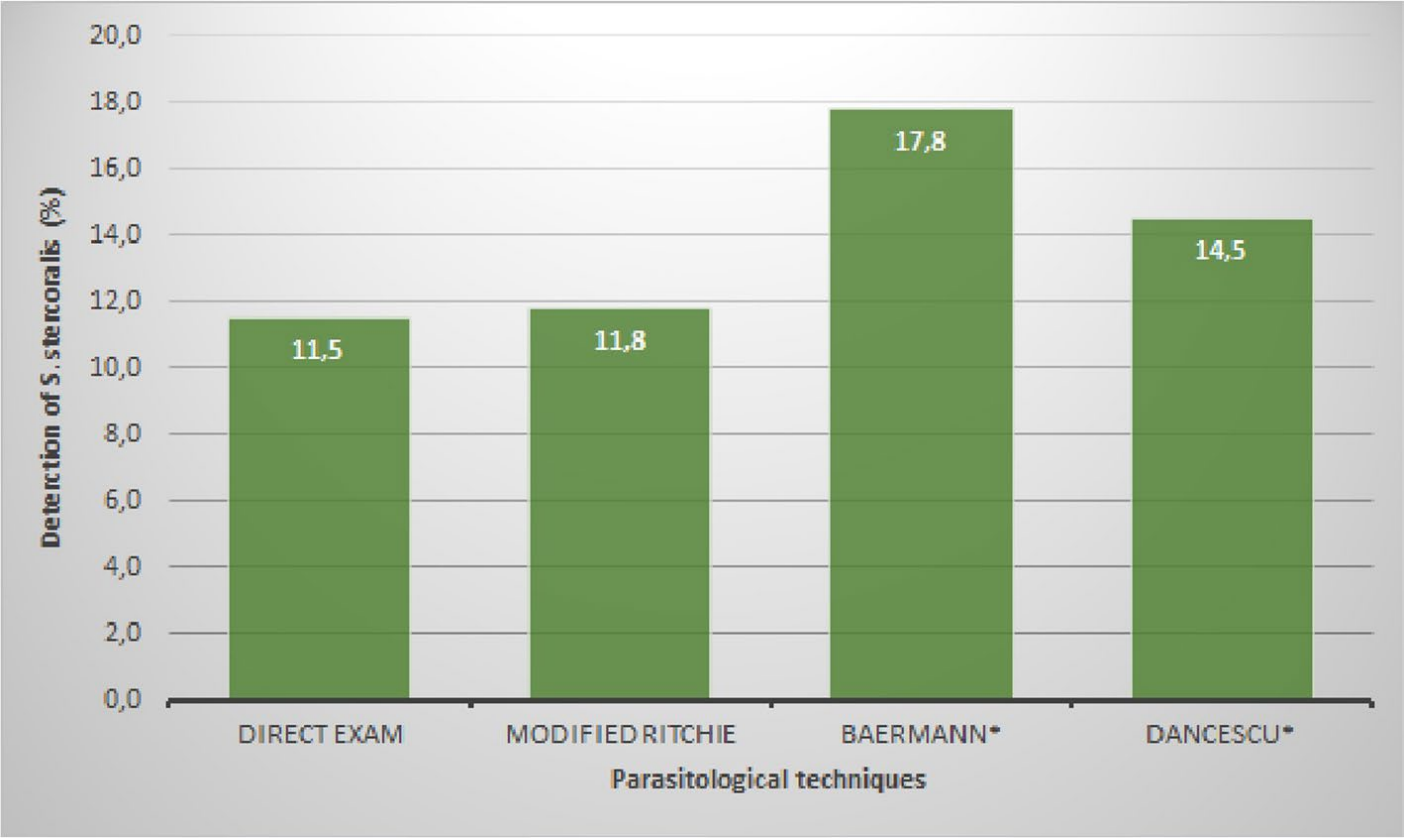
Detection of *Strongyloides stercoralis* by four parasitological techniques, in primary health care in children from Bolivian tropical areas. * Specific methods for *Strongyloides stercoralis*

The performances of these techniques, specifically for the detection of *S. stercoralis*, showed some differences (Fig. 3). Among them, the Baermann technique, which detects 17.8% (N: 54) of positive samples, is higher than the 14.5% (N: 44) found by the Dancescu technique, higher and significant compared to the direct exam or Ritchie technique (Chi^2^ test, p < 0.01 in both cases). While the direct examination (11.5%, N: 35) and modified Ritchie method (11.8%, N: 36) found a similar percentage much lower than the specific ones.

Given the better performance of the Baermann technique, to compare the sensitivity and specificity, Baermann was used as the standard for the detection of *S. stercoralis* in our study, since there is no other better technique in Bolivian PHC (Table 1).

**Table 1:** Sensitivity and specificity of parasitological techniques in the detection of *Strongyloides stercoralis* using Baermann technique as the standard

Technique	Sensitivity (95%CI)	Specificity (95%CI)	PPV (95%CI)	NPV (95%CI)	Accuracy (95%CI)	*(K)*
Direct exam	62.9% (48.7– 75.7)	99.6% (97.8 – 99.9)	97.2% (82.9 – 99.6)	92.5% (89.6 – 94.5)	93.1% (89.5 – 95.6)	0.73*
Dancescu	75.9% (62.4 – 86.5)	98.8% (96.5 – 99.7)	93.3% (81.7 – 97.7)	94.2% (92.1 – 96.8)	94.7% (91.5 – 96.9)	0.81*
Modified Ritchie	66.7% (52.5 – 78.9)	99.7% (98.5 – 99.9)	100% (NA – NA)	93.2% (90.4 – 95.2)	94% (90.7 – 96.4)	0.77*

**Not**: (K) inter-rater reliability or Cohen’s kappa coefficient; * = *p* < 0.05. *PPV* Positive Predictive Value, *NPV* Negative Predictive Value

The highest sensitivity with respect to the Baermann technique was only for the Dancescu technique (75.9%), followed by the Modified Ritchie technique (66.7%) which is used at the second-line care and with lower sensitivity for S. stercoralis the direct examination, which reached 62.9%. In terms of accuracy and a high Cohen’s kappa coefficient, only the Dancescu technique showed the highest concordance with Baermann, as it is a specific method for *S. stercoralis*. However, the concordance of the direct examination (K: 0.73) and the modified Ritchie technique (K: 0.77) was equally good, showing better results with the one applied at the second-line care.

## Discussion

The present study aimed to demonstrate the applicability of a simple, low-cost, and specific method for improving the detection of *S. stercoralis*. Based on our results, the Baermann method should be considered one of the best parasitological options to increase diagnostic sensitivity in the PHC system of Bolivia, not only because of its better sensitivity than direct examination but also because of its low cost and easy implementation with few adaptations, as demonstrated in this and other studies [24–26].

The presence of STHs, such *as S. stercoralis*, is associated with a lack of basic sanitary conditions in the home area, which is linked to poverty. In addition, these infections cause children’s physical growth and intellectual development to become stunted, which, in the long term, is reflected in their diminished capacity for work and productivity. This is the main reason the World Health Organization (WHO) urges the search for strategies to improve the monitoring, evaluation, and treatment of parasites as part of a program to control them, especially in high-risk areas such as the tropics [27] .

It is difficult to control and prevent *S. stercoralis* infection in public health without proper diagnosis [28]. For this, the use of the Baermann technique would be an approach to solve this problem at the first-line in PHCs of endemic regions, whose precarious conditions do not allow the implementation of complex or expensive methods [24]. The Baermann technique would constitute a useful diagnostic tool and a first step for the control of *S. stercoralis* infection, because it increases the sensitivity of the currently used methods in the Bolivian system. Compared to other techniques, such as Dancescu, reviewed in this study, it does not require complex materials or specialised health personnel. Although it cannot compete with other techniques with better results, its implementation would be easier, faster, and less costly for the public health system with short-term results, as in other countries in the region [9, 10, 29]. It is expected that with the implementation of the new universal health insurance “SUS”, which is being implemented by the Bolivian government, the school-age child population will have access to early diagnosis and prompt treatment for these infectious diseases [30].

However, *S. stercoralis* is accompanied by other pathogenic species, such as *G. duodenalis* and hookworms, owing to environmental conditions in tropical areas and similar entrance doors, as in the case of hookworms [31]. Therefore, the use of direct examination remains an ideal and low-cost method in first-line units of the Bolivian PHC system to examine multiparasitism, which, in association with the Baermann technique, would increase the sensitivity of S. stercoralis detection for early treatment and prevention of its spread in vulnerable areas [32].

There is still a lack of research in the country and region on the effects of persistent parasitic infections, such as those produced by *S. stercoralis*, on development during childhood, which, in addition to being under-diagnosed, are not sufficiently controlled or treated [33, 34]. Therefore, the Bolivian health system still faces important challenges in terms of intervention policies for the prevention and control of STHs, particularly in tropical areas, owing to the environmental and social conditions [1, 14]. Another limitation of the new parasitological techniques is the low possibility of identifying other species in the Strongyloidae group. Especially in animals that live at home with the family, such as dogs, this has been one of the risk factors significantly associated with the presence of *S. stercoralis* in children in this study [35–37]. This study did not consider the application of other techniques, such as agar-plate culture or serological techniques, because of their complex development, more specific and costly materials, and special training of health personnel, which would represent a difficulty for the long-term sustainability of these services in the Bolivian PHC system. Another important element to consider about the Baermann technique is that it does not require lengthy or expensive training of personnel; our team that developed the technique in the present study required less than one month to master the technique.

In conclusion, to improve the early diagnosis and treatment of *Strogyloides stercoralis* infections, it is important to consider the patient’s socio-environmental factors and habits, especially in children, because the symptoms and signs may not be clear in the first stage. With a high suspicion of the presence of this parasite associated with its factors, the use of the Baermann technique is strongly recommended in addition to direct examination at the first-line in the primary health care system. In addition, the Baermann technique is easy to implement in the basic laboratory of a first-line unit because of its simplicity and low cost, making it a promising candidate for improving the diagnosis of *S. stercoralis* in the tropical zones of Bolivia and similar regions.

## Supplementary Information

**Additional file 1.** DATASET CBBA_SS: file containing the digitised database of the questionnaires and laboratory records coded.

## Abbreviations

PHC: Primary Health Care
PP: Pathogenic Parasite
*S. stercoralis*: *Strongyloides stercoralis*
STH: Soil Transmitted Helminth
SUS: (from Spanish) Universal Health Insurance
